# Differentiating founder and chronic HIV envelope sequences

**DOI:** 10.1371/journal.pone.0171572

**Published:** 2017-02-10

**Authors:** John M. Murray, Stephen Maher, Talia Mota, Kazuo Suzuki, Anthony D. Kelleher, Rob J. Center, Damian Purcell

**Affiliations:** 1 School of Mathematics and Statistics, UNSW Sydney, Sydney, New South Wales, Australia; 2 Zuse Institute Berlin, Berlin, Germany; 3 Department of Microbiology and Immunology, Peter Doherty Institute for Infection and Immunity, University of Melbourne, Melbourne, Victoria, Australia; 4 The Kirby Institute, UNSW Sydney, Sydney, New South Wales, Australia; Universidad Autonoma de Madrid Centro de Biologia Molecular Severo Ochoa, SPAIN

## Abstract

Significant progress has been made in characterizing broadly neutralizing antibodies against the HIV envelope glycoprotein Env, but an effective vaccine has proven elusive. Vaccine development would be facilitated if common features of early founder virus required for transmission could be identified. Here we employ a combination of bioinformatic and operations research methods to determine the most prevalent features that distinguish 78 subtype B and 55 subtype C founder Env sequences from an equal number of chronic sequences. There were a number of equivalent optimal networks (based on the fewest covarying amino acid (AA) pairs or a measure of maximal covariance) that separated founders from chronics: 13 pairs for subtype B and 75 for subtype C. Every subtype B optimal solution contained the founder pairs 178–346 Asn-Val, 232–236 Thr-Ser, 240–340 Lys-Lys, 279–315 Asp-Lys, 291–792 Ala-Ile, 322–347 Asp-Thr, 535–620 Leu-Asp, 742–837 Arg-Phe, and 750–836 Asp-Ile; the most common optimal pairs for subtype C were 644–781 Lys-Ala (74 of 75 networks), 133–287 Ala-Gln (73/75) and 307–337 Ile-Gln (73/75). No pair was present in all optimal subtype C solutions highlighting the difficulty in targeting transmission with a single vaccine strain. Relative to the size of its domain (0.35% of Env), the α_4_β_7_ binding site occurred most frequently among optimal pairs, especially for subtype C: 4.2% of optimal pairs (1.2% for subtype B). Early sequences from 5 subtype B pre-seroconverters each exhibited at least one clone containing an optimal feature 553–624 (Ser-Asn), 724–747 (Arg-Arg), or 46–293 (Arg-Glu).

## Introduction

There has been a significant global effort to develop an effective vaccine for HIV. Vaccine trials to date have shown limited efficacy but what success there has been was associated with the ability of the vaccine to stimulate HIV envelope glycoprotein (Env) antibodies [1, 2]. Hence future vaccine candidates will most likely include a component that elicits antibodies specific to targets on Env. However, HIV-1 has an extremely high rate of sequence evolution and strains in different communities form distinct subtypes [3]; high strain diversity exists between individuals and even within any one patient [4]. The challenge for vaccines stimulating antibodies to Env is to target rare common epitopes between viral strains, and ideally between subtypes [1]. These targets should be representative of early founder virus clones that emerge through the strain-selecting bottleneck of transmission [5].

A newly infecting clone quickly undergoes sequence evolution impacted by a developing immune response, as assessed by differences between founder and chronic viral sequences [5–11]. Importantly for vaccines aiming to prevent transmission, HIV infection generally results from a single transmitted virus strain [5, 8], indicating the potential benefits of identifying key transmission-related features. Envelope features are of particular relevance as it is the only HIV protein exposed on the outer surface of an infectious particle, also making it the target for neutralising antibodies [12]. Despite extreme Env sequence diversity, all strains must preserve the functional properties of CD4 receptor binding and binding to chemokine coreceptors such as CCR5 and CXCR4 that facilitate entry. Almost all founder virus uses the CCR5 coreceptor [11], a trait mostly encoded in the V3 loop of Env [13]. Other HIV Env functional domains include motifs allowing interaction with cell adhesion and trafficking receptors, like the Integrin α_4_β_7_ glycoprotein. This glycoprotein acts as a gut-homing receptor for lymphocytes, targeting them to the extensive gut associated lymphatic tissues (GALT) that are important sites for explosive viral expansion in the early phase of infection [14–16].

Envelope is the target for broadly neutralizing antibodies (bNAb), and several conserved tertiary structures on Env have been identified as vulnerable sites for bNAb binding [17, 18]. It is unclear whether important new conformational targets for bNAb might be selected during transmission. Understanding what Env features are unique to the transmitted virus but which evolve to escape selection under immune pressure, may point to possible vaccine targets [11, 19].

One approach to identifying vaccine targets is to determine individual amino acids (AA) that differ significantly between chronic and founder sequences among the 857 positions of the Env glycoprotein gp160 [7]. This glycoprotein is cleaved to form the 511 AA CD4 binding subunit gp120, and the 346 AA gp41 that is required for fusion of the virus with the cell membrane. This non-covalently bound heterodimer self-associates into trimers that form the functional Env spike on the viral surface that determines viral tropism. By chance there will be many differences in these sequences, given the large variability in some regions of Env. Functionally related sites can also be compared between these groups, as well as the number of glycosylation sites [7, 9]. This direct comparison of individual positions or known functional sites has proven useful but is limited. How AA and regions within the linear Env genetic sequence determine function is related to their positions in the complex 3-dimensional Env trimer structure [20–22], and determining the interplay between AA in this structure is not straightforward. Any susceptibility in the transmission virus will result in the Env sequence evolving in a series of compensatory escape mutations, so that the trimer structure is altered in order to avoid or minimise the effectiveness of the developing immune response [11, 12]. This collection of escape mutations need not be contiguous in the sequence but will form a biologically related network of positions in Env.

One way of identifying biologically related areas within a highly structured protein involves calculating positions on a set of AA sequences that covary. Pairs of positions are said to covary if the AA combinations observed at these positions are sufficiently different from random combinations. For example if in 16 Env sequences at positions 223 and 432 there were 8 Phe-Lys pairs and 8 Tyr-Arg pairs then positions 223 and 432 would covary since these observed pairs are sufficiently different to the random combinations of 4 Phe-Lys, 4 Phe-Arg, 4 Tyr-Lys and 4 Tyr-Arg pairs. It would also suggest that these positions are linked in some functional manner. If a virus is to evolve under immune pressure, then a single mutation is usually insufficient and a number of compensatory, fitness-restoring mutations at other positions are required [23, 24]. Each of these compensatory mutations results in a covarying pair and the entire set of mutations results in a network of covarying pairs [25]. Analysing sequences using the covariance between amino acid positions is a useful approach in a variety of applications [26–29]. In the case of Env, amino acid sequence networks of covarying pairs contain possible vaccine targets, especially if we can identify those combinations that are transmission signatures present in founder sequences but absent in chronic sequences. Here we follow the approach developed in Murray et al. [30], where optimisation methods were used in combination with covariance calculations to determine the most prominent features that differentiate one hepatitis C virus group from another. We apply these methods to sets of founder and chronic HIV subtype B and subtype C Env sequences, with the aim of identifying features that distinguish founder from chronic virus and that represent possible vaccine targets.

## Results

As described previously [9], HIV Env sequences for 133 transmission strain cases (78 subtype B, 55 subtype C) were obtained from Keele et al. and Abrahams et al. [5, 8]. Subtype C is the most common virus in Africa whereas subtype B is prevalent in developed countries such as Australia and the US. These founder sequences represent the inferred single clones that gave rise to productive infection in these individuals [5, 8]. A comparison group of 133 HIV Env sequences were derived from the plasma of individuals with chronic infection, obtained from the Los Alamos National Laboratory (LANL) HIV sequence database. Each randomly selected control sequence was investigated and submitted to rigorous exclusion criteria. Controls were frequency-matched on HIV-1 subtype and geographical location (consistent with the approach of Gnanakaran et al. [7]), with 78 subtype B chronic sequences selected from USA/Trinidad and Tobago and 55 subtype C sequences selected from South Africa/Malawi.

**Fig 1:**
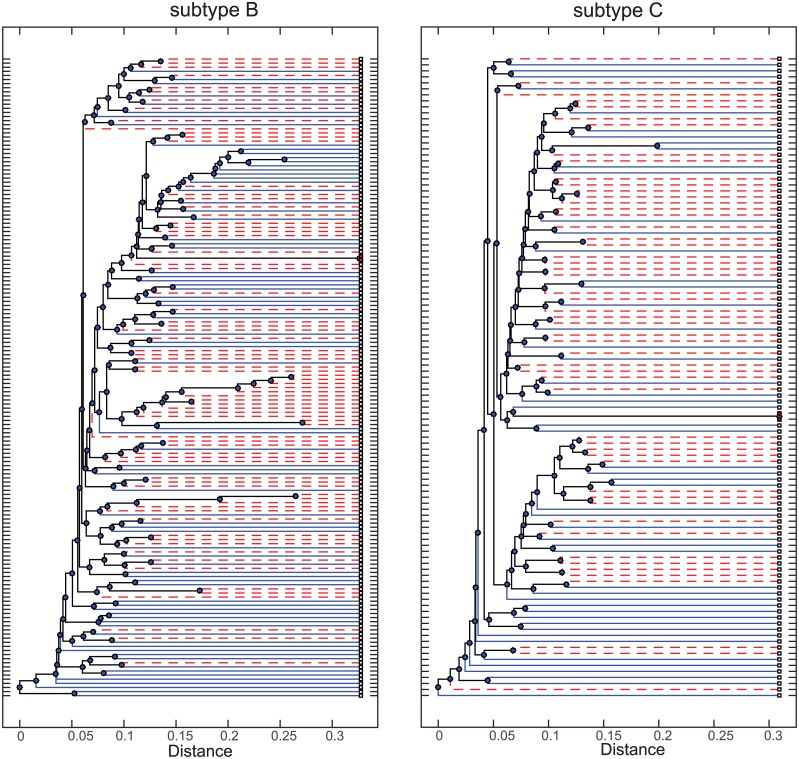
Unrooted phylogenetic trees for the subtype B and C HIV Env sequences. Founder sequences are shown with red dashed branches, while chronic sequences are denoted by blue branches.

The sequences were aligned and numbered relative to the HXB2 reference strain [31]. The phylogenetic trees for these subtypes and how the Founder and Chronic sequences distribute are shown in Fig 1.

**Fig 2:**
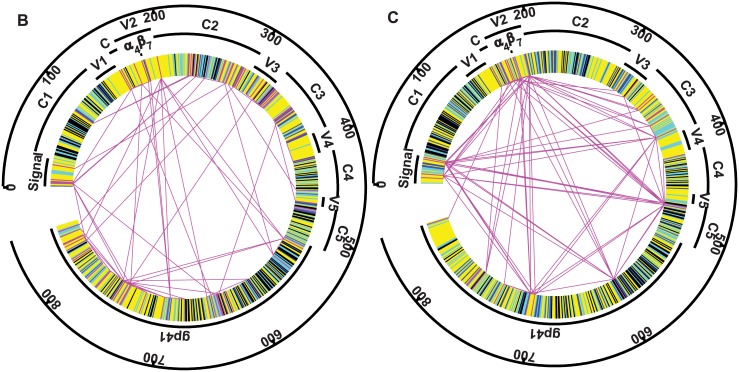
Conserved and covarying regions for subtypes B and C *Env*. Regions are coloured as conserved across both subtypes (black), conserved within each subtype (dark blue), conserved except for a maximum of 2 individuals in that subtype (light blue), and covarying (magenta). Those covarying pairs with at least 20% of the maximum covariance value for subtype C, and 12.5% for subtype B, are connected with magenta lines. The different levels of covariance were determined to include approximately the same numbers of covarying pairs in each case: 78 for subtype B and 79 for subtype C. The signal, α_4_β_7_ binding site, constant (C1-C6) and variable (V1-V5) regions within gp120, and the gp41 domain are mapped onto the *Env* sequence.

After alignment of the approximately 857 long AA Env sequences, covarying pairs were calculated as previously described [25, 30], to indicate AA positions in Env that are possibly connected through function, where a change in one position is likely to also result in a change in another position. In this study we analyse both subtype B and C viruses, hence it was necessary to perform the covariance calculations separately for each subtype. There were 2,495 covarying pairs over the 156 subtype B sequences and 3,021 subtype C covarying pairs over the 110 subtype C sequences. The maximum covariance values were similar for each of the subtypes, 14.0 for subtype B and 14.6 for subtype C, however there were more covarying pairs for subtype C that were close to this maximum value (Fig 2). The slightly higher covariance among subtype C, despite fewer sequences, tends to reflect the more clustered phylogenetic tree. This has been observed previously with differences in covariance between 1a and 1b HCV sequences mirroring the clustering of these sequences [30].

Covarying AA contain possible targets for a vaccine-stimulated response since they are not totally conserved, but are not so variable as to represent random changes. We mapped regions that were conserved, covarying and variable in *Env* over both subtypes (Fig 2). For subtype C, highly covarying pairs clustered in the signal, V2, and C5 regions of gp120 and in gp41. The pair with the highest covariance was 474–476, while the positions that appeared most were 192 (22 times), 476 (12), 27 (12), 11 (11), 706 (11), 388 (10), 595 (10). Covarying pairs were incident (one of the positions within the pair) 11 times to the α_4_β_7_ binding site (positions 179 to 181). For subtype B the pair with the highest covariance was 230–232, connecting within an N-linked glycosylation site (NXS/T at positions 230, 231, 232), and the other highly covarying pairs tended to be in close proximity as well. One of these covarying pairs (181–693), was also incident to the α_4_β_7_ binding site.

### Optimal networks

The covarying pairs determine components within the sequences that vary but not in a random fashion. However as depicted in Fig 2, there can be many of these. Our aim was to determine what is special in founder viruses. We define a *separating* pair as a covarying pair of positions and its AA combination, where at that pair of positions, the AA combination is exhibited by some sequence in the founder group but by none in the chronic group. Hence we search among separating pairs to determine signatures of founder virus that are not expressed by any chronic virus in our samples. To determine those that are most pertinent to characterising founder virus we calculated optimal collections of separating pairs. Optimality was based on requiring the fewest pairs to separate these groups or that tended to maximise covariance (Methods). Separation of Founders from Chronics becomes more difficult as fewer pairs are used. Hence the solution with the fewest pairs will likely contain stronger predictors of what differs between these groups. This is similar in essence to identifying AA and their positions where comparisons between the groups gives the lowest p or q values [7]. Initially a single optimal network was identified for each of the subtype/covariance group combinations (covariance over all, founder, or chronic sequences), and objective function type (fewest pairs or optimising covariance). Hence, twelve optimal networks were identified and used in this part of the analysis.

Optimization enforces two limitations on this problem. Firstly it can only be applied to a single alignment rather than a bootstrapped collection of alignments. To compensate for this limitation we only included covariance calculated over positions that contained at most 10% gaps. This had the effect of excluding some of the more variable regions around indels. Secondly this binary integer programming problem belongs to the class of Non-Polynomially (NP) Hard problems that are characterised by small increases in problem size (here this is related to the number of covarying pairs) resulting in large increases in computational time. Partly for this reason we limited the size of the problem by only including pairs with a covariance value of at least 0.5, leading to the 2,495 covarying pairs for subtype B described above. Including pairs with a covariance of 0.1 would have led to an almost 10-fold increase in the number of pairs (21,937 for subtype B). The difficulty of solving these problems with lower covariance cut-off is highlighted by the related *connected* optimal network problem being computationally intractable in reasonable time even with the 0.5 cut-off [32]. Nevertheless to address sensitivity to this assumption, we also investigate in a later section a slightly simpler problem but with no covariance cut-off.

The optimal networks, determining features of Founder sequences that separate them from Chronic sequences, required between 13 and 15 AA pairs for subtype B (S1 Table). The fewer subtype C sequences required between 11 and 13 AA pairs to separate all Founder sequences from Chronic sequences. Some of the pairs within the optimal networks appeared multiple times for the 6 optimization problems in each subtype/group combination (3 minimizing the number of pairs, and 3 optimizing a measure of total covariance). For subtype B the pairs 278–620 (Ser Asp), and 750–836 (Asp Ile) each appeared 4 times, while for subtype C the most frequently appearing pairs (3 times each) were 170–192 (His Arg) and 588–662 (Arg Ala) (Table 1).

**Table 1:** Pairs observed multiple times (frequency f) in optimal networks for each subtype. The number of individuals exhibiting each AA combination is denoted by n.

B					C				
AA positions		f	n	AA	AA positions		f	n	AA
278	620	4	15	SD	170	192	3	5	HR
750	836	4	14	DI	588	662	3	6	RA
230	232	3	3	DQ	7	10	2	7	QY
232	236	3	10	TS	161	192	2	4	AI
535	620	3	9	LD	179	674	2	4	PN
151	178	2	3	GN	192	343	2	5	IQ
240	340	2	8	KK	295	334	2	7	EN
283	621	2	3	IE	344	346	2	7	KG
291	792	2	4	AI	352	379	2	8	YG
293	337	2	5, 3	VD, QK	393	727	2	4	DP
319	836	2	6	TT	417	770	2	8	QQ
336	845	2	9	ET	448	727	2	6	SL
347	543	2	10	TL	721	727	2	7	IL
440	620	2	5	KD					
624	747	2	8	ER					
724	758	2	10	RD					
724	837	2	11	RF					
747	758	2	3	QD					
818	840	2	11	IF					

Several single positions within these Env sequences appeared frequently in optimal networks (Table 2). Positions 836 and 620 for subtype B, and positions 192 and 346 for subtype C were the most frequently appearing single positions. Position 346 for subtype C was also proximal to position 347 appearing 6 times for subtype B in the constant C3 region of gp120. The next most frequently appearing proximal positions were 178 for subtype B and 179 for subtype C that were within or next to the α_4_β_7_ binding site.

**Table 2:** Single AA in optimal networks determined on Founders, which appear at least 2 times (frequency f). The region of Env is denoted ahead of a decimal point, and any recognised motif after the decimal.

B			C		
position	domain	f	position	domain	f
836	41CT.LLP-1 α helix	10	192	V2	8
620	41ED.HR2	9	346	C3	7
232	C2.NGS	6	727	41CT.KenEpi	6
347	C3	6	624	41ED	5
336	C3	5	7	Sig	4
535	41ED.aHR1	5	161	V2	4
724	41CT	5	179	V2.α_4_β_7_	4
747	41CT	5	295	V3.NGS(2G12)	4
750	41CT.NGS	5	588	41ED.HR1	4
178	V2.α_4_β_7_	4	10	Sig	3
230	C2	4	170	V2	3
278	C2	4	344	C3	3
291	C2.NGS	4	350	C3	3
621	41ED	4	393	V4	3
624	41ED	4	662	41ED	3
758	41CT	4	674	41ED	3
92	C1.120•41	3	721	41CT	3
151	V1	3	832	41CT.LLP-1 α helix	3
236	C2.NGS	3	27	Sig	2
240	C2.NGS	3	29	Sig	2
283	C2	3	172	V2	2
293	C2	3	181	V2.α_4_β_7_	2
319	V3.R5/X4bs	3	334	C3	2
354	C3	3	337	C3	2
543	41ED	3	343	C3	2
837	41CT.LLP-1 α helix	3	352	C3	2
24	Sig	2	379	C3	2
181	V2.α_4_β_7_	2	417	C4	2
335	C3	2	440	C4	2
337	C3	2	448	C4	2
340	C2.NGS	2	496	C5	2
440	C4	2	619	41ED	2
444	C4	2	621	41ED	2
553	41ED	2	770	41CT.LLP-2 α helix	2
640	41ED	2	833	41CT.LLP-1 α helix	2
792	41CT.LLP-3 α helix	2			
818	41CT	2			
833	41CT.LLP-1 α helix	2			
840	41CT.LLP-1 α helix	2			
845	41CT.LLP-1 α helix	2			

### Multiple optimal solutions for each subtype/group combination

The optimization procedure above determines an optimal solution but these are not unique. For example, the first row of S1 Table shows that an optimal solution for the problem where we minimize the number of pairs, contains 13 pairs for subtype B and 11 pairs for subtype C, when covariance is calculated over all sequences within each subtype. However a total of 13 different optimal networks that each differ by at least one AA pair for subtype B can be identified for this particular problem (Table 3). Similarly, for the subtype C sequences there are 75 optimal solutions. However these optimal solutions share several features. For subtype B there are 9 pairs that are present in each of the 13 optimal solutions, indicating possibly susceptible immune-evasion pathways. For subtype C, 644–781 (Lys Ala) is present in 74 solutions, while 2 pairs appear in 73 solutions, and 6 pairs in 72 solutions. It is interesting to note that there are no pairs for subtype C that are present in all 75 optimal solutions, suggesting a greater number of pathways by which this virus evolves and possibly making it a more difficult vaccine target.

**Table 3:** The pairs that are observed in a given number of optimal solutions for subtypes B and C. Listed for each of the optimal separating pairs are the covarying positions, the amino acids for the sequences in the Founder separating pairs, and then the Env motifs for each of these positions. The optimization problem was solved using objective i) (minimizing the number of pairs) on a covariance network constructed using all sequences in each subtype.

**Subtype B (13 optimal solutions)**			
*Observed in 13 solutions*			
178–346	Asn Val	V2.α_4_β_7_[33]	C3
232–236	Thr Ser	C2.NGS	C2.NGS
240–340	Lys Lys	C2.aNGS	C3
279–315	Asp Lys	C2.CD4bs[34, 35]	V3.R5/X4bs[36]
291–792	Ala Ile	C2.NGS	41CT.LLP-3 α helix[37–39]
322–347	Asp Thr	V3.R5/X4bs[36]	C3
535–620	Leu Asp	41ED.aHR1	41ED.HR2
742–837	Arg Phe	41CT.KenEpi[40]	41CT.LLP-1 α helix[37–39]
750–836	Asp Ile	41CT.NGS	41CT.LLP-1 α helix[37–39]
*Observed in 11 solutions*			
92–346	Lys Val	C1.120•41	C3
*Observed in 10 solutions*			
588–836	Lys Thr	41ED.HR1	41CT.LLP-1 α helix[37–39]
**Subtype C (75 optimal solutions)**			
*Observed in 74 solutions*			
644–781	Lys Ala	41ED.HR2	41CT.LLP-2 α helix[37–39]
*Observed in 73 solutions*			
133–287	Ala Gln	V1 hvr	C2.nCD4bs[34, 35]
307–337	Ile Gln	V3.R5/X4bs[36]	C3
*Observed in 72 solutions*			
10–346	Tyr Gly	Sig	C3
132–841	Ser Leu	V1 hvr	41CT.LLP-1 α helix[37–39]
295–322	Glu Asp	V3.NGS(2G12)[41]	V3.R5/X4bs[36]
721–727	Ile Leu	41CT	41CT.KenEpi [40]
778–779	Val Val	41CT.LLP-2 α helix[37–39]	41CT.LLP-2 α helix[37–39]
779–833	Val Val	41CT.LLP-2 α helix[37–39]	41CT.LLP-1 α helix[37–39]

The region of Env is denoted ahead of a decimal point, and any recognised motif after the decimal. The covarying pairs are separated by—a dash. Sig = Env signal peptide; C2 = constant domain 2; C3 = constant domain 3, V1 = variable domain 1, V2 = variable domain 2, V3 = variable domain 3, 41ED = gp41 ectodomain external to membrane; 41CT = gp41 cytoplasmic tail internal to the membrane; α_4_β_7_ = alpha-4-beta-7 integrin binding site; NGS = N-linked glycosylation site; aNGS = amino acid adjacent to NGS; nNGS = near to NGS; C3 = constant region 3.; CDbs = residues mapped to contacting at the CD4 binding site; R5/X4bs = residues mapped to contact R5 or X4 coreceptor; V1hvr = Variable region 1 hyper variable region; HR1 = helix region 1; aHR1 = adjacent to HR1; HR2 = helix region 2 that contains T20 drug site; 120•41 contact residues between gp120 and gp41; KenEpi = Kennedy Epitope–highly immunogenic epitope [40]; LLP-1 helix = lentiviral lytic peptide– 1 alpha helix; LLP-2 helix = lentiviral lytic peptide– 2 alpha helix; LLP-3 helix = lentiviral lytic peptide– 3 alpha helix.

### Optimal networks with no covariance restriction

By restricting pairs to those with a covariance value of 0.5 or higher, we attempted to determine functionally relevant positions. However this excluded many other possibilities. Allowing all pairs to be considered in separating Founder from Chronics, increased the number of pairs more than 50-fold, making the calculation of optimal networks with criterion (ii) that incorporated the covariance value computationally impractical. However we could still determine optimal networks that achieved separation of Founders from Chronics with the fewest number of pairs. The optimal subtype B network contained 12 separating pairs: 46–293 (Arg Glu), 84–333 (Val Val), 269–767 (Asp Lys), 278–620 (Ser Asp), 291–758 (Ala Val), 293–375 (Val Thr), 336–535 (Glu Met), 345–842 (Ile Asn), 535–624 (Lys Asp), 553–624 (Ser Asn), 724–747 (Arg Arg), 750–836 (Asp Ile). Despite including many more pairs, this optimal network contained only one or two fewer optimal pairs than with the above S value cut-off. Moreover 750–836 (Asp Ile) appeared in all multiple optimal solutions (Table 3).

The subtype C optimal solution without covariance restriction consisted of 9 pairs: 9–515 (Asn Met), 12–350 (Gln Ser), 166–352 (Lys His), 330–620 (Tyr Thr), 352–379 (Tyr Gln), 448–821 (Ser Ala), 565–588 (Met Arg), 640–778 (Asn Val), 833–837 (Leu Cys).

### Occurrence of optimal pairs in virus cloned prior to seroconversion

The above calculations determined aspects of founder sequences that might be targeted by vaccines. Given the large number of covarying pairs determined in this way, it is unlikely that all of these pairs would be robust predictors of susceptibility. To test this we sequenced subtype B Env virus from 5 individuals who were newly infected (Methods). The optimal pairs (for all subtype B solutions in S1 Table) showed little overlap with the clones derived from these pre-seroconverters (Table 4). Part of this limitation was due to the cloning process only including positions 44 to 752. Three of the five individuals and 5 of the 12 clones exhibited some of the optimal pairs but these were limited to: 343–621 (Gln Asp) and 354–636 (Pro Asp) for one clone; 553–624 (Ser Asn) for 2 individuals; 624–747 (Glu Arg) for 2 individuals, and 724–747 (Arg Arg) for one individual.

**Table 4:** Comparison of optimal Founder pairs with the AA combinations appearing for 5 subtype B pre-seroconverters with each of the clones sequenced.

Founder pairs		Env motifs connected	Patient: PSC35		PSC89	PSC24		PSC73					PSC182	
			Clone: 5	10	51	948	955	911	912	913	914	915	928	949
343–621	QD	aNGS–aHR2	DD	HE	QE	DD	ED	QE	***QD***	EY	IE	QE	HQ	KQ
354–636	PD	aNGS -HR2	PS	PS	PS	PN	PS	PN	***PD***	PS	PS	PN	NS	GN
553–624	SN	HR1- NGS	***SN***	SD	S-	SE	SD	NE	SD	SG	***SN***	SD	SG	NN
624–747	ER	NGS—aNGS	NR	DR	-R	***ER***	DR	***ER***	DR	GR	NR	DR	GR	NR
724–747	RR	KenEpi- aNGS	PR	PR	PR	***RR***	PR	PR	PR	PR	PR	PR	QR	PR
46–293	RE		RR	RQ	***RE***	KE	KE	RK	RK	KE	KE	KA	***RE***	KE

The two pairs 553–624 (Ser Asn) and 724–747 (Arg Arg) were also in the optimal network determined for subtype B when there was no covariance restriction. After including the 46–293 (Arg Glu) optimal pair, these three features were exhibited by at least one clone from each seroconverter (Table 4).

## Discussion

**Fig 3:**
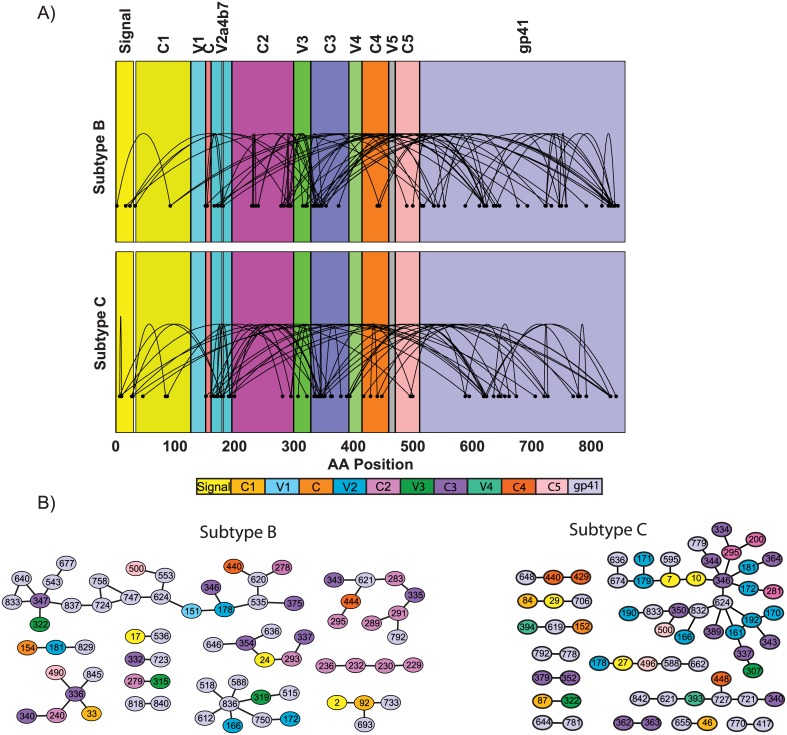
The collection of optimal pairs displayed relative to the domains of Env. A) over a linear representation and B) as networks.

By searching over covarying pairs that exhibit covariance above a minimal background level, we are attempting to determine aspects of Env that exhibit some change, and hence are likely to reflect, and to be susceptible to, an evolving immune response. Optimal pairs, selected either by being part of a network with the fewest pairs, or maximizing a measure of covariance, in some sense represent fundamental aspects of what separates Founder from Chronic virus, since separation cannot be achieved using fewer pairs or with a higher measure of covariance. This is analogous to determining AA with the lowest p or q values in a comparison at individual positions of Env [7]. As with these statistically significant AA, the optimal pairs may indicate components within transmitted viruses that determine selection at the transmission bottleneck and that can be targeted by bNAbs. Although there are a large number of different optimal pairs when collected over all problems (60 subtype B and 54 subtype C, determined from the unique pairs contained within S1 Table), these are not randomly distributed over Env (Fig 3). The majority are incident to gp41 (54 and 36 pairs respectively have at least one position in gp41), and occur at regions related to viral fusion and at AA internal to the membrane involved in virion structure and intracellular signalling. There are 20 and 22 optimal pairs respectively contained entirely within gp120 and that are incident to the C3 region, which supports the view that these calculations are not entirely driven by the variable regions. While the next highest region with incident pairs in subtype B is the C2 region (19 pairs) this was not frequent for subtype C (5 pairs) which instead had high incidence in V2 (19 pairs, 7 pairs for subtype B). Several identified covarying founder virus sites beyond 665 exist within or beneath the membrane layer and would not directly bind antibody.

The gut-homing α_4_β_7_ integrin has been implicated in higher susceptibility to HIV infection [42] and α_4_β_7_ high CD4+ T cells have been suggested as preferential targets in mucosal transmission especially for subtype C [15]. The viral α_4_β_7_ binding site is located within V2 at positions 179 to 181. Despite only covering 3 of the 857 AA in Env, optimal pairs incident to this domain were particularly prevalent but especially so for subtype C: 4.2% of optimal pairs (S1 Table) relative to 0.35% of Env and a ‘*prevalence ratio*’ of 11.9 (4.2%/0.35%). Of interest is that several α_4_β_7_ binding site optimal pairs were also incident to the external fusion or membrane internal domains of Env gp41. While structural connections between V2 and the gp41 fusion domains can be envisioned from the crystal and cryo-EM structures of Env gp160 [20, 22], these are not anticipated across the membrane, and suggest a functional connection, possibly in cell-cell viral transfer. The remainder of V2 was also prevalent in these optimal pairs for subtype C with a prevalence ratio of 4.6. The α_4_β_7_ binding site was less likely to be represented in this analysis for subtype B with a prevalence ratio of 3.3, but still higher than the other domains. However these results are in contrast to findings that blocking binding to α_4_β_7_ had little impact on infection by founder subtype C virus [43], or that HIV Env is in general a poor ligand for this integrin [44]. Nevertheless monoclonal antibodies that targeted α_4_β_7_ integrin-expressing CD4+ T cells protected rhesus macaques from SIV infection, possibly suggesting a large role played by early virus in binding to these cells [45], although it is unclear whether this effect was through altered Env binding and entry, or through altered gut mucosal trafficking. The subtype C α_4_β_7_ pairs were also incident to the domains: signal (1), V2 (1), C3 (2) and gp41 (2). Of note is that the pairs between α_4_β_7_ and C3, 181–346 (Val Gln) and 181–364 (Ile Ala), are incident to N-linked glycosylation sites (339–341 or 344–346, 362–364). Glycosylation sites in C3 have been observed to affect α_4_β_7_ reactivity [15].

AA within gp41 or the α2 helix in C3 (positions 335 to 353), tended to be central to the networks of all optimal pairs for each subtype (Fig 3B), exhibiting the greatest node degrees within those networks. In particular the C3 α2 helix positions formed a strongly connected subnetwork for subtype C that also included V2 and α_4_β_7_ positions, suggesting these Founder characteristics evolved under immune pressure in a related manner. This is consistent with observations of the staged development of antibodies targeting the α2 helix of C3 followed by those targeting V1-V2 for subtype C [46].

We identified all optimal solutions relative to the problem of minimizing the number of pairs with covariance over all sequences, there being many more for subtype C than for subtype B (Table 3) possibly related to the higher covariance network exhibited by subtype C (Fig 2) and perhaps reflecting a more diverse pathway of evolution for this genotype. Every subtype B optimal solution contained a core set of 9 covarying pairs some of which have been identified through their interactions with bNAbs. Glycans at positions 234 and 276 are essential for the reactivity of bNAb 8ANC195 [18], where these would be impacted by linked mutations at nearby sites in the optimal pairs 232–236 and 279–315, that lie at the gp120-gp41 ectodomain structural interface [20, 22, 47]. Mutations at positions 89, 90, 227, 232 and 243 also diminished neutralization by bNAb 35O22 [48]. Ten of the 39 founder sequences contained the 232–236 Thr Ser combination, while none of the chronic sequences displayed this feature, revealing potential differences in N-linked glycosylation that may optimise the viral entry functions involving the gp120-gp41 interface, but expose a neutralisation sensitive epitope.

As may be expected by the much larger number of optimal solutions for subtype C, there were no pairs that were common to all solutions. This would suggest that there may be no single Env target that will be effective in blocking transmission to an individual in a community where subtype C is prevalent. Investigation of glycans for an elite subtype C neutralizer showed N611D and N637K as well as E647A had the greatest effect on neutralization which may be relevant to the 644–781 pair appearing in 74/75 optimal solutions [49]. That the covarying AA lie beneath the membrane and are shielded from antibody access, raises the possibility of either structural alterations across the membrane, or effects on cell-cell transmission involving intracellular molecular interactions. One of the glycosylation sites that varied across subtype C virus commenced at position 133 [50], part of the optimal pair in 73 of 75 optimal solutions. Positions on the α2 helix of gp120: 236, 305, 332, 335, 336, 337, 343, 350 and 393 are strongly selected in subtype C for resistance to NAb [51], which may be relevant to the appearance in multiple optimal solutions of 10–346 Tyr Gly (72/75) and 307–337 Ile Gln (73/74).

Testing whether these optimal subtype B Founder pairs were also represented in early Env sequences from 5 pre-seroconverters showed little overlap: only 2 pairs connecting C3 to gp41, and three within gp41 with 2 of these involving gp41 sequence inside the membrane (Table 4). However one strength of this analysis is that the pairs that did overlap with the seroconverter sequences were not contained in any of the 78 Chronic sequences. Only positions 44 to 752 of Env were sequenced in the cloning process which therefore omitted comparison with some of the optimal pairs. These pre-seroconverter clones may also be variants from the virus that established the infections approximately 20 days earlier. Effects on cell signalling or cell-cell transmission may have promoted the identification of AA located beneath the membrane. Nevertheless identified pairs incident to glycosylation sites were most likely to play a role with these occurring in the pre-seroconverters at or near positions 354 in C3 and 624 in gp41. A comparison of the seroconverter sequences with the 12 optimal pairs determined for subtype B with no covariance restriction revealed 3 features that were exhibited by at least one clone in each individual: 553–624 (Ser Asn), 724–747 (Arg Arg) (these two were identified in the above analysis) and 46–293 (Arg Glu).

The optimization and comparison calculations determining differences between Founders and Chronics can only be performed on a single alignment. Given the high degree of variability and the presence of indels in these sequences there will be a number of possible alignments with differences occurring mainly at, or adjacent to, gaps in the alignment. Partly because of this we omitted positions where more than 10% of sequences contained gaps or were uncertain. Including these positions leads to optimal separating networks more highly incident to indels (data not shown) and as such less likely to be robust targets for vaccine stimulated antibodies. Omitting positions that were predominantly gaps meant that we were less able to investigate differences in length of some Env regions between Founders from Chronics as previously observed by us and others [9, 52]. However the number of positions omitted were not markedly different between Founders vs Chronics—for subtype B there were 26 positions in the Founder sequences and not in Chronics consisting entirely of gaps and 18 positions in Chronics but not in Founders (20 vs 17 respectively for subtype C). Dropping positions also excluded 10 of the 31 HXB2 glycosylation sites so that our analysis will not be able to use changes in their number as a factor, but which we had previously analysed [9].

A further limitation of our approach is that these sequences were not matched for factors apart from subtype and geographical region. Matching Founders and Chronics by gender, transmission mode, etc., would assist in removing extraneous components that confound separation due to vaccine-relevant factors, but it would reduce the power of the analysis and the generality of the results. Our general approach of searching among covarying positions, also assumes that mutation away from the transmitted virus occurs along a few pathways in response to immune pressure and that then induce covariance. This is generally true for the mutational pathways within HIV resulting from the development of drug resistance to a particular antiretroviral agent. Founder envelope sequences have also been observed to co-evolve with broadly neutralizing antibody [12, 46], which may indicate that initially susceptible features in Env can be deduced from the covariance induced by the resulting mutational pathways. However all covariances within Env need not be related to changes due to immune pressure so that the differences we determine between Founders and Chronics can encompass aspects that will not be relevant to vaccine targets.

The covarying pairs link regions within Env. Linkage across domains, which is not surprising given the complex trimer structure which brings variable and conserved regions into close proximity [22], is known to impact on infectivity and function [53, 54]. Some of the linkages determined here may be due to the similar geographical regions from which the sequences originated. Although chronic sequences were frequency-matched by geographical location, this cannot be completely ruled out as contributing to some aspects of the optimal networks determined here. The optimal pairs are likely components of more diverse networks that may more precisely describe the multiple binding sites of any bNAbs or the mutational pathways that the virus follows to evade them. The effects measured on gp41 AA beneath the membrane could also arise from emergence of T cell responses. How these larger networks can be extracted from this analysis is a more complex problem. It would also be beneficial to investigate these in silico results in an *in vitro* setting.

In summary we have used operations research methods to determine the most prominent features of Env that differ between founder and chronic sequences for subtype B and subtype C. Our results suggest that the gut-homing α_4_β_7_ integrin plays a role in establishing infection and may indicate key AAs desirable for inclusion in vaccine strains. Unlike subtype B where 9 AA pairs were in all optimal solutions, no single AA pair was present in all subtype C optimal solutions (Table 3). This may highlight difficulties in targeting transmission with a single vaccine strain, especially for subtype C.

## Materials and methods

Founder and chronic DNA sequences, as well as the HXB2 reference envelope sequence [55], were converted to AA sequences (nt2aa, MATLAB 2012b, The MathWorks Inc., Natick MA, USA), and then aligned using a progressive multiple alignment method (multialign). Pairwise distances were calculated with the Jukes-Cantor method, with the phylogenetic tree generated using the Unweighted Pair Group Method Average (seqlinkage). AA positions in the aligned sequences were numbered relative to HXB2 according to the convention of Korber et al. [31].

### Pre-seroconverters

Env was sequenced from an additional set of subtype B virus for 5 newly infected males (estimated 20 to 23 days from transmission, Fiebig Stage II (n = 4), and III (n = 1)) [56], where transmission was through men having sex with men. Due to the cloning process only positions 44 to 752 of Env were sequenced from these transmission HIV strains.

These pre-seroconverter individuals were enrolled in a naturally history cohort study, the Primary HIV and Early Disease Research: Australian Cohort (PHAEDRA), that was established by the National Centre in HIV Epidemiology and Clinical Research to monitor immunological and virological characteristics of individuals with acute and early HIV-1 infection. Research ethics approval (number 02244) was given by St Vincent's Hospital, Sydney, Research Ethics Committee. All participants signed an informed consent form before study entry.

### Networks

The construction of a covariance network was previously described by Murray et al. [30], such that the nodes of the network are given by each AA position, and each pair of covarying positions above the cut-off value provides an edge. Subtype B and C sequences were investigated separately, along with three different sequence groups: i) all sequences within the subtype (All), ii) the founder sequences and iii) the chronic sequences. As such, for a given subtype and sequence group all covarying positions are contained in the set *Psubtype*,*group*. A network was then constructed for each subtype and sequence group combination. We calculated optimal networks based on each of these sets, extracting the “best” separating pairs (a separating pair is defined as a covarying pair of positions and an AA combination present in at least one of the founder sequences but in none of the chronic sequences), according to certain criteria and where each founder sequence contained at least one of these separating pairs. The optimal separation of Founders from Chronics was performed using two criteria: i) separating with the fewest pairs, and ii) using a weighting that simultaneously minimized the number of pairs and maximized a measure of total covariance. The latter objective was achieved by applying the weight *w*_*k*_ *= (Ŝ-S*_*k*_*)*^*2*^ to the cost of pair *k*, where *Ŝ* is the integer ceiling of the maximum of all covariance values.

For these calculations we considered separating pairs for each AA combination so that optimal solutions will extract single AA combinations at each pair of positions in the optimal network [30].

As an example, the problem *P*_*B*,*All*_ with optimal criterion (i) determines the smallest set of AA combinations expressed by Founder sequences at covarying pairs where these were calculated on all subtype B sequences. In this instance, an optimal solution consists of the set of 13 pairs and AA described by {2–92 (Arg Lys), 24–293 (Ile Lys), 166–836 (Arg Thr), 178–535 (Asn Val), 232–236 (Thr Ser), 240–340 (Lys Lys), 279–315 (Asp Lys), 291–792 (Ala Ile), 322–347 (Asp Thr), 535–620 (Lys Asp), 612–836 (Ala Lys), 724–837 (Arg Phe), 750–836 (Asp Ile)}, where each Founder sequence contains at least one (and possibly more) of these features, while no Chronic sequence exhibits any of these features. In this way this set of AA pairs separates all Founders from all Chronics. The optimality aspect guarantees that there are no other combinations that separate the groups with fewer combinations, although there may be other sets with the same number of pairs and hence are also optimal.

The binary integer programming method used to extract this smallest set from all separating pairs (formulated as described in Murray et al. [30]), is a standard optimization procedure, where a variable *x*_*i*_ is assigned to the *i*^th^ separating pair and given the value 1 if it is included in the optimal set and value 0 otherwise. For this problem optimality is determined by allocating the fewest 1 values among the *x*_*i*_.

### Multiple optimal solutions for each subtype and group combination

These problems determining optimal separating pairs do not necessarily have unique solutions. Multiple optimal solutions were identified using an iterative approach. Initially, the problem was solved to identify an optimal solution. A constraint was then added to the problem to exclude the current solution. The problem was then resolved to identify another optimal solution. For example, after calculating the optimal solution above, the separating pair 2–92 (Arg Lys) was excluded and the problem resolved. The solution to this restricted problem also contained 13 pairs, and so comprised an additional optimal solution. On the other hand, excluding 750–836 (Asp Ile) resulted in a solution requiring 14 pairs to separate the two groups and so any optimal solution must include this combination.

### Optimal solutions with no lower bound on covariance

The optimal solutions above were calculated over all pairs with a covariance value of at least 0.5. This implied that the pairs might exhibit some functional relationship between them otherwise the AAs at these positions would more likely combine in a random manner. Different cut-off values would allow more or fewer covarying pairs which would impact the optimal solutions. To determine how much this could change we calculated optimal networks, only using criterion (i) that minimized the number of pairs, over all pairs regardless of covariance. The size of these problems, 177,906 subtype B pairs and 157,641 subtype C pairs, made calculating the optimal solution with criterion (ii), impractical for this NP Hard problem. As long as we are not interested in generating connected networks where the AA match at the connecting position [32], we can reduce the number of separating pairs by only including those that are not dominated by others—a separating pair is said to be *dominant* if it is expressed by a set of Founders *I*_*G*_ and where there is no other separating pair which is expressed by Founders *I*_*G’*_ such that *I*_*G*_ ⊆ *I*_*G’*_., For example if the separating pair 180Ser-230Asn is exhibited by Founders 1, 2 and 3 while 130Ala-485Tyr is exhibited by Founders 1, 2, 3 and 5, then the first pair is excluded from the calculations of separating Founders from Chronics with the fewest number of pairs. The pair 130Ala-485Tyr *dominates* 180Ser-230Asn. After this calculation there are only 1,358 dominant pairs for subtype B Founders and 928 dominant pairs for subtype C. The binary integer programming method above can then be applied to this problem with no covariance restriction.

All calculations were performed with Matlab version R2012b (The MathWorks Inc., Natick MA, USA). The binary integer programming problems were solved using the bintprog routine and the CPLEX toolbox (IBM, Armonk NY, USA).

## Supporting information

S1 Table Pairs of AA in optimal networks that separate Founders from Chronics. Each item lists the pairs in the optimal network when calculations are performed over covariance calculations determined on sequences in All, Founders or Chronics (Sep. Set). These are features exhibited by some founder sequences but by no chronic sequence. The number of sequences that exhibit that feature for that AA pair are listed as (n). Optimality was determined either through choosing the fewest number of pairs (Prob **=** No) or through maximizing a measure of total covariance (Prob **=** Yes).
(DOCX)
