# Supplementary material for: Differentiating founder and chronic HIV envelope sequences
Source: PLoS One. 2017 Feb 10;12(2):e0171572. doi: 10.1371/journal.pone.0171572 (PMC5302377; doi:10.1371/journal.pone.0171572)
Supplement: S1 Table — Each item lists the pairs in the optimal network when calculations are performed over covariance calculations determined on sequences in All, Founders or Chronics (Sep. Set). These are features exhibited by some founder sequences but by no chronic sequence. The number of sequences that exhibit that feature for that AA pair are listed as (n). Optimality was determined either through choosing the fewest number of pairs (Prob = No) or through maximizing a measure of total covariance (Prob = Yes). (DOCX) [file pone.0171572.s001.docx]

# Differentiating founder and chronic HIV envelope sequences

John M Murray^1*^, Stephen Maher^1,2^, Talia Mota^3^, Kazuo Suzuki^4^, Anthony D Kelleher^4^, Rob J Center^3,#^, Damian Purcell^3^

^1^ School of Mathematics and Statistics, UNSW Australia, Sydney, NSW 2052, Australia

^2^ Zuse Institute Berlin, Takustr. 7, 14195 Berlin, Germany

^3^ Department of Microbiology and Immunology, University of Melbourne, Australia

^4^ The Kirby Institute, UNSW Australia, Sydney, NSW 2052, Australia

^#^ Current address: Centre for Biomedical Research, Burnet Institute, Melbourne, Australia

* Corresponding author:

Email: j.murray@unsw.edu.au

## Supporting Table S1

Pairs of AA in optimal networks that separate Founders from Chronics. Each item lists the pairs in the optimal network when calculations are performed over covariance calculations determined on sequences in All, Founders or Chronics (Sep. Set). These are features exhibited by some founder sequences but by no chronic sequence. The number of sequences that exhibit that feature for that AA pair are listed as (n). Optimality was determined either through choosing the fewest number of pairs (Prob=No) or through maximizing a measure of total covariance (Prob=Yes).

| Sep. Set | Prob | Subtype B | | Subtype C | |
| --- | --- | --- | --- | --- | --- |
|  |  | Pairs (n) | Features | Pairs (n) | Features |
| All | No | 2-92 (6)  24-293 (5)  166-836 (5)  178-535 (3)  232-236 (10)  240-340 (8)  279-315 (5)  291-792 (4)  322-347 (7)  535-620 (9)  612-836 (4)  724-837 (11)  750-836 (14) | RK  IK  RT  NV  TS  KK  DK  AI  DT  LD  AL  RF  DI | 152-619 (4)  166-832 (8)  171-179 (4)  172-281 (7)  179-674 (4)  181-346 (3)  181-364 (4)  190-833 (8)  346-624 (4)  417-770 (8)  588-662 (6) | EY  KV  QS  AV  PN  VG  IA  EL  SE  QQ  RA |
|  | Yes | 154-181 (3)  178-346 (6)  229-230 (3)  230-232 (3)  232-236 (10)  240-340 (8)  289-291 (3)  291-335 (4)  293-337 (5)  347-833 (4)  444-621 (3)  535-620 (9)  588-836 (5)  724-837 (11)  750-836 (14) | ML  NV  NQ  DQ  TS  KK  KY  TE  VD  TL  NK  LD  KT  RF  DI | 7-10 (7)  7-179 (3)  161-192 (4)  161-624 (6)  170-192 (5)  295-334 (7)  344-346 (7)  350-832 (4)  362-363 (4)  393-621 (6)  429-440 (3)  721-727 (7) | QY  LP  AI  TE  HR  EN  KG  KA  NQ  GD  RA  IL |
| Founder | No | 151-178 (3)  240-336 (5)  278-620 (15)  283-335 (8)  291-792 (4)  336-490 (8)  347-543 (10)  347-640 (8)  375-535 (10)  535-620 (9)  553-624 (8)  724-747 (12)  818-840 (11) | GN  KE  SD  TS  AI  TQ  TL  EG  TM  LD  SN  RR  IF | 7-10 (7)  179-674 (4)  200-295 (8)  295-346 (5)  307-337 (7)  344-346 (7)  350-500 (6)  352-379 (8)  393-727 (4)  448-727 (6)  778-792 (8) | QY  PN  TE  NS  IQ  KG  AK  YG  DP  SL  VI |
|  | Yes | 17-536 (8)  33-336 (8)  92-693 (4)  151-178 (3)  151-624 (4)  230-232 (3)  232-236 (10)  278-620 (15)  293-337 (3)  347-543 (10)  500-553 (7)  543-677 (6)  640-833 (8)  747-758 (3)  818-840 (11) | GA  QE  KV  GN  EN  DQ  TS  SD  QK  TL  RS  LQ  NV  QD  IF | 10-346 (7)  170-192 (5)  192-343 (5)  295-334 (7)  350-833 (7)  352-379 (8)  393-727 (4)  440-648 (5)  448-727 (6)  621-842 (6)  624-832 (9) | YG  HR  IQ  EN  KV  YG  DP  AK  SL  EH  GV |
| Chronic | No | 92-733 (7)  172-750 (3)  278-620 (15)  283-621 (3)  295-444 (3)  319-836 (6)  336-845 (9)  343-621 (4)  347-837 (5)  354-636 (3)  440-620 (5)  518-836 (6)  624-747 (8)  724-758 (10)  750-836 (14) | KI  EA  SD  IE  TR  TT  ET  QD  TF  PD  KD  LV  ER  RD  DI | 29-706 (5)  46-655 (4)  161-337 (6)  172-346 (5)  192-624 (6)  344-779 (4)  394-619 (4)  417-770 (8)  496-588 (4)  588-662 (6)  721-727 (7) | RN  KN  AK  VG  IE  KT  SQ  QQ  VQ  RA  IL |
|  | Yes | 24-354 (3)  181-829 (5)  230-232 (3)  278-620 (15)  283-621 (3)  319-515 (4)  319-836 (6)  332-723 (6)  336-845 (9)  354-646 (4)  440-620 (5)  624-747 (8)  724-758 (10)  747-758 (3)  750-836 (14) | IP  LI  DQ  SD  IE  AM  TT  TA  ET  EI  KD  ER  RD  QD  DI | 7-595 (3)  27-178 (5)  27-496 (3)  29-84 (7)  87-322 (3)  161-192 (4)  170-192 (5)  192-343 (5)  340-721 (6)  389-624 (6)  588-662 (6)  636-674 (6)  644-781 (9) | QM  LK  MI  SM  VD  AI  HR  IQ  EI  KG  RA  DS  KA |
